# Basal late sodium current is a significant contributor to the duration of action potential of guinea pig ventricular myocytes

**DOI:** 10.14814/phy2.13295

**Published:** 2017-05-29

**Authors:** Yejia Song, Luiz Belardinelli

**Affiliations:** ^1^ University of Florida Gainesville Florida; ^2^ Former employee of Gilead Sciences, Inc. Foster City California

**Keywords:** Action potential duration, Ca^2+^/calmodulin‐dependent protein kinase II, late sodium current, Na_V_1.5 channel, ventricular myocytes

## Abstract

In cardiac myocytes, an enhancement of late sodium current (*I*_N__aL_) under pathological conditions is known to cause prolongation of action potential duration (APD). This study investigated the contribution of *I*_N__aL_ under basal, physiological conditions to the APD. Whole‐cell *I*_N__aL_ and the APD of ventricular myocytes isolated from healthy adult guinea pigs were measured at 36°C. The *I*_N__aL_ inhibitor GS967 or TTX was applied to block *I*_N__aL_. The amplitude of basal *I*_N__aL_ and the APD at 50% repolarization in myocytes stimulated at a frequency of 0.17 Hz were ‐0.24 ± 0.02 pA/pF and 229 ± 6 msec, respectively. GS967 (0.01–1 *μ*mol/L) concentration dependently reduced the basal *I*_NaL_ by 18 ± 3–82 ± 4%. At the same concentrations, GS967 shortened the APD by 9 ± 2 to 25 ± 1%. Similarly, TTX at 0.1–10 *μ*mol/L decreased the basal *I*_NaL_ by 13 ± 1–94 ± 1% and APD by 8 ± 1–31 ± 2%. There was a close correlation (*R* ^2^ = 0.958) between the percentage inhibition of *I*_N__aL_ and the percentage shortening of APD caused by either GS967 or TTX. MTSEA (methanethiosulfonate ethylammonium, 2 mmol/L), a Na_V_1.5 channel blocker, reduced the *I*_NaL_ by 90 ± 5%, suggesting that the Na_V_1.5 channel isoform is the major contributor to the basal *I*_NaL_. KN‐93 (10 *μ*mol/L) and AIP (2 *μ*mol/L), blockers of CaMKII, moderately reduced the basal *I*_NaL_. Thus, this study provides strong evidence that basal endogenous *I*_NaL_ is a significant contributor to the APD of cardiac myocytes. In addition, the basal *I*_NaL_ of guinea pig ventricular myocytes is mainly generated from Na_V_1.5 channel isoform and is regulated by CaMKII.

## Introduction

The late Na^+^ current (*I*_NaL_) is a component of the fast inward *I*_Na_, which remains activated during the plateau and the repolarization of a cardiac action potential (Noble and Noble 2006; Antzelevitch et al. 2014). *I*_NaL_ is increased in congenital and acquired pathological conditions, such as long QT syndrome type 3, cardiac hypertrophy, heart failure, and myocardial ischemia (Belardinelli et al. 2015; Makielski 2016). An enhancement of *I*_NaL_ under these pathological conditions may cause a prolongation of the action potential duration (APD) and is considered potentially arrhythmogenic (Belardinelli et al. 2015; Makielski 2016). Inhibition of *I*_NaL_ by *I*_NaL_ blockers, such as ranolazine, has shown promising antiarrhythmic value (Belardinelli et al. 2015; Makielski 2016). However, because the amplitude of *I*_NaL_ under physiological conditions is relatively small, its role (i.e., the *I*_NaL_ in the absence of drug or pathological modification) in cardiac repolarization has not been fully recognized.

Several lines of evidence suggest that the inward *I*_NaL_ may play a significant role in maintaining cardiac depolarization under physiological conditions. (1) *I*_NaL_ can remain activated throughout the action potential plateau, where the membrane resistance is high (Weidmann 1951). Therefore, even a small net inward current may cause a significant lengthening of the plateau and thus the APD. (2) The APD is shortened in the presence of TTX, an inhibitor of *I*_NaL_ (Coraboeuf et al. 1979; Kiyosue and Arita 1989). (3) In canine ventricular myocytes, the density of *I*_NaL_ is greater in the mid‐myocardium, compared with that in the epi‐ and endomyocardium (Zygmunt et al. 2001). In keeping with that, TTX‐induced APD shortening is greater in the mid‐myocardium than in the epi‐ and endomyocardium (Zygmunt et al. 2001). (4) In failing hearts of both human and canine models, inactivation of *I*_NaL_ of ventricular myocytes is further slowed, compared with that in myocytes of normal hearts (Maltsev et al. 2007). The enhanced *I*_NaL_ contributes to the prolongation of APD (Maltsev et al. 2007), and conversely, inhibition of *I*_NaL_ by ranolazine shortens the APD of ventricular myocytes isolated from a canine heart failure model (Undrovinas et al. 2006).

The goal of this study was to determine the contribution of basal *I*_NaL_ to the APD of ventricular myocytes of healthy guinea pigs. In the past, a precise evaluation of the contribution of basal *I*_NaL_ to the APD has been hindered by the small amplitude of the current and the lack of a selective inhibitor. Most studies of *I*_NaL_ have been conducted in the presence of *I*_NaL_ enhancers, such as anemone toxin II (ATX‐II) (Isenberg and Ravens 1984; Song et al. 2004). In this study, the selective *I*_NaL_ inhibitor GS967 (Belardinelli et al. 2013) and low concentrations of TTX were applied to selectively block *I*_NaL_. The amplitude of *I*_NaL_ in this study was not preenhanced by drugs or special experimental conditions, except for one series of experiments in which the *I*_NaL_ enhancer ATX‐II was applied to verify the specificity of the action of GS967. Thus, the subject of this study was cardiac *I*_NaL_ under basal conditions. The role of basal *I*_NaL_ in maintaining the depolarization of the ventricular action potential was assessed by comparing the percentage inhibition of *I*_NaL_ with the percentage shortening of APD. In addition, we examined whether the basal *I*_NaL_ is generated from Na_V_1.5 channels, and whether the basal *I*_NaL_ is regulated by Ca^2+^/calmodulin‐dependent protein kinase II (CaMKII), respectively, by applying the selective Na_V_1.5 channel blocker MTSEA (methanethiosulfonate ethylammonium) (Haufe et al. 2005; O'Reilly and Shockett 2012) and the CaMKII inhibitors KN‐93 and AIP (autocamtide‐2‐related inhibitory peptide).

## Materials and Methods

Animal use was approved by the Institutional Animal Care and Use Committee, and conformed to the *Guide for the Care and Use of Laboratory Animals* (National Research Council, 2011). Hearts of guinea pigs of either sex were isolated and perfused via the aorta with warm (35°C) and oxygenated solutions in the following order: (1) Tyrode solution containing (in mmol/L) 135 NaCl, 4.6 KCl, 1.8 CaCl_2_, 1 MgCl_2_, 10 glucose, and 10 HEPES, pH 7.4, for 5 min; (2) Ca^2+^‐free solution containing (in mmol/L) 100 NaCl, 30 KCl, 2 MgCl_2_, 10 glucose, 10 HEPES, 15 taurine, and 5 pyruvate, pH 7.4, for 5 min; and (3) Ca^2+^‐free solution containing collagenase (120 units/mL) and albumin (2 mg/mL), for 20 min. At the end of the perfusion, the ventricles were minced and gently shaken for 10 min in the collagenase solution to release single cells. Only the quiescent myocytes with clear striations were used for this study.

Transmembrane voltages and currents were recorded using the whole‐cell patch‐clamp technique. Data were acquired and analyzed with an Axopatch‐200 amplifier, a Digidata‐1440A digitizer, and pCLAMP‐10 software. All experiments were performed at 36°C.

For measurements of action potentials, cells were incubated in the Tyrode solution (bath solution). The recording pipettes were filled with a solution containing (in mmol/L) 120 K‐aspartate, 20 KCl, 1 MgSO_4_, 4 Na_2_ATP, 0.1 Na_3_GTP, and 10 HEPES, pH 7.3. A depolarizing pulse was applied every 6 sec to elicit action potentials. The APD was determined from the beginning of depolarization to the time when 30% (APD_30_), 50% (APD_50_), and 90% (APD_90_) of repolarization were completed.

For measurements of *I*_NaL_, myocytes were superfused with a bath solution containing (in mmol/L) 135 NaCl, 1.8 CaCl_2_, 1 MgCl_2_, 10 glucose, 10 HEPES, 4.6 CsCl, 0.05 NiCl_2_, and 0.01 nitrendipine, pH 7.4. The recording pipettes were filled with a solution containing (in mmol/L) 120 Cs‐aspartate, 20 CsCl, 1 MgSO_4_, 4 Na_2_ATP, 0.1 Na_3_GTP, and 10 HEPES, pH 7.2. Sodium current was activated by 200–250 msec long voltage‐clamp pulses applied every 10 sec, from a holding potential of −90 mV to a test potential of −30 or −50 mV. The amplitude of *I*_NaL_ was calculated as the average amplitude of current during the last 100 msec of a depolarizing pulse.

GS967 was synthesized by Gilead Sciences. MTSEA was purchased from Toronto Research Chemicals, KN‐93 and KN‐92 from Calbiochem, AIP from Tocris, and ATX‐II from Sigma. KN‐93, KN‐92, and AIP were applied through the recording pipette solution; other drugs were added to the bath solutions. The duration of each drug treatment was 3 min before recording.

Data are expressed as mean ± SEM. Sample size (*n*) is shown as number of cells/from number of hearts. Statistical analyses were conducted using SigmaPlot software. Concentration–response relationship and EC_50_ for GS967 inhibition of *I*_NaL_ were calculated from a standard four‐parameter logistic curve fitted with the following equation:

$$y=\min+\frac{\max-\min}{1+{\left(\frac{x}{\mathrm{EC}50}\right)}^{-\mathrm{Hillslope}}}$$

Coefficient of determination (*R* ^2^) was calculated from a standard linear regression curve fitted with the following model:

$$f=y^{0}+a^{*}x$$

The *t*‐test or one‐way ANOVA followed by Holm–Sidak method was applied for statistical analysis. A *P* < 0.05 was considered statistically significant.

## Results

### Contribution of basal *I*_NaL_ to APD

**Figure 1:**
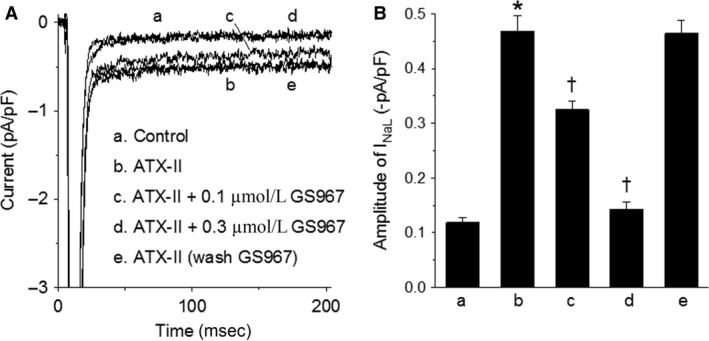
Concentration‐dependent inhibition by GS967 of ATX‐II (5 nmol/L)‐induced *I*_N__aL_. Inward currents were activated by depolarizing pulses from −90 to −50 mV. Panel A, superimposed currents recorded in the order of a–e from a single myocyte before (control) and after drug treatments. Panel B, summary of the average amplitude of *I*_N__aL_ recorded before (A) and after (B–E) drug treatments, as shown in panel A (*n* = 12/5). *P < 0.001 versus control; ^†^ *P* < 0.001 versus ATX‐II alone.

To verify the action of GS967 as an *I*_NaL_ blocker, the effect GS967 on *I*_NaL_ induced by the *I*_NaL_ enhancer ATX‐II was examined. In this series of experiments, *I*_NaL_ was activated by voltage‐clamp pulses from −90 to −50 mV. ATX‐II (5 nmol/L) increased the amplitude of *I*_NaL_ at −50 mV from −0.12 ± 0.01 to −0.47 ± 0.03 pA/pF (*n* = 24/9, *P* < 0.001). GS967 reversibly and concentration dependently inhibited the *I*_NaL_ in the presence of ATX‐II. GS967 at concentrations of 0.1, and 0.3 *μ*mol/L significantly (*P* < 0.001, *n* = 12/5) reduced the amplitude of ATX‐II‐stimulated *I*_NaL_ by 41 ± 2% and 93 ± 5%, respectively (Fig. 1). In another group of myocytes (*n* = 12/4), the ATX‐II‐stimulated *I*_NaL_ was inhibited by 0.03 and 1 *μ*mol/L GS967 by 24 ± 3% and 100%, respectively (*P* < 0.001, not shown).

**Figure 2:**
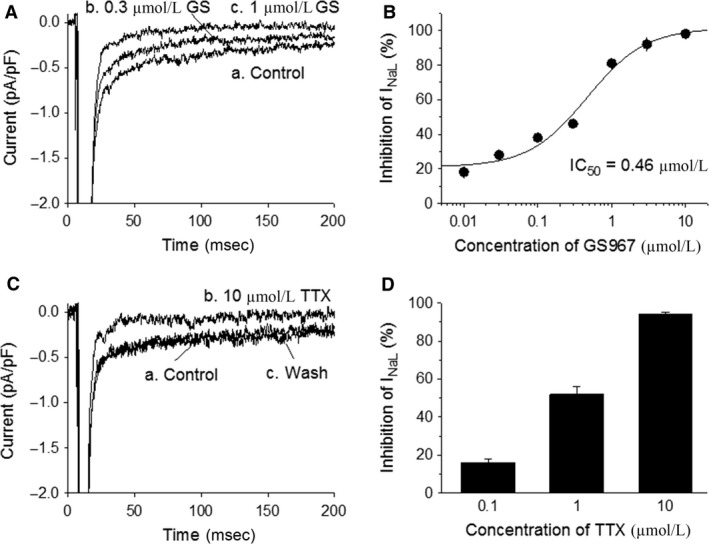
Concentration‐dependent inhibition by GS967 or TTX of basal *I*_N__aL_. *I*_N__aL_ was elicited by voltage‐clamp pulses from −90 to −30 mV. Panel A, example of current traces recorded from a single myocyte in the absence of drugs (control) and in the presence of 0.3 and 1 *μ*mol/L GS967 (GS). Panel B, concentration–response relationship of the inhibitory effect of GS967 on *I*_N__aL_. Each data point represents an average inhibition observed from 10 myocytes isolated from 3 to 5 hearts. Data points are fitted with a four‐parameter logistic curve. Panel C, current traces recorded before (A) and after (B) application of TTX, and after washing out TTX (C). Panel D, bars show an average inhibition of *I*_N__aL_ by 0.1 (*n* = 13/4), 1 (*n* = 13/4), and 10 (*n* = 18/6) *μ*mol/L TTX, respectively.

To estimate the amplitude of basal *I*_NaL_, voltage‐clamp pulses from −90 to −30 mV were applied to activate inward *I*_Na_. The average amplitude of *I*_NaL_ at −30 mV was −0.24 ± 0.02 pA/pF (*n* = 40/17). GS967 at concentrations of 0.01, 0.03, 0.1, 0.3, 1, 3, and 10 *μ*mol/L, respectively, concentration dependently reduced the amplitude of basal *I*_NaL_ by 18 ± 3, 28 ± 3, 38 ± 2, 46 ± 2, 82 ± 4, 91 ± 4, and 100% (*P* < 0.05, *n* = 10/3–5 for each concentration; Each myocyte was treated with 2–3 concentrations of GS967), with an IC_50_ of 0.46 *μ*mol/L (Fig. 2, A and B). TTX at concentrations of 0.1, 1, and 10 *μ*mol/L, respectively, significantly (*P* < 0.001) decreased the amplitude of *I*_NaL_ by 16 ± 2% (*n* = 13/4), 52 ± 4% (*n* = 13/4), and 94 ± 1% (*n* = 18/6; Fig. 2C and D), further confirming that the *I*_NaL_ was indeed an inward sodium current.

**Figure 3:**
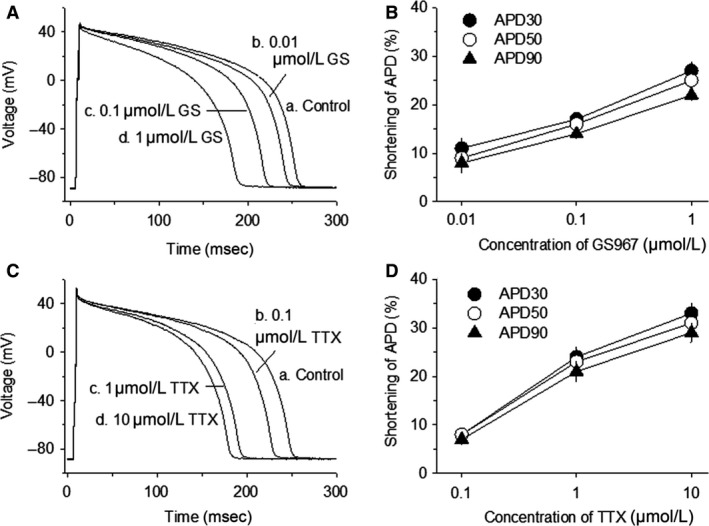
Concentration‐dependent shortening by GS967 or TTX of the action potential duration (APD). Panel A, example of action potential traces recorded from a single myocyte before (control) and after applications of 0.01, 0.1, and 1 *μ*mol/L GS967 (GS). Panel B, summary of APD shortening caused by GS967 at concentrations of 0.01 (*n* = 10/4), 0.1 (*n* = 25/10), and 1 (*n* = 19/7) *μ*mol/L, respectively. Panel C, action potentials recorded from a myocyte in the absence of drug (control) and in the presence of 0.1, 1, and 10 *μ*mol/L TTX. Panel D, average shortening of APD caused by TTX at concentrations of 0.1 (*n* = 9/2), 1 (*n* = 15/4), and 10 (*n* = 15/3) *μ*mol/L.

The baseline APD_30_, APD_50_, and APD_90_ measured from the myocytes were 198 ± 5 msec, 229 ± 6 msec, and 248 ± 6 msec, respectively (*n* = 43/14). GS967 at concentrations of 0.01 (*n* = 10/4), 0.1 (*n* = 25/10), and 1 (*n* = 19/7) *μ*mol/L, respectively, significantly (*P* < 0.003) shortened the APD_30_ by 11 ± 2, 17 ± 1, and 27 ± 2%, APD_50_ by 9 ± 2, 16 ± 1, and 25 ± 1%, and APD_90_ by 8 ± 2, 14 ± 1, and 22 ± 1% (Fig. 3, A and B). TTX at concentrations of 0.1(*n* = 9/2), 1 (*n* = 15/4), and 10 (*n* = 15/3) *μ*mol/L significantly (*P* < 0.05) decreased the APD_30_ by 8 ± 1, 24 ± 2, and 33 ± 2%, APD_50_ by 8 ± 1, 23 ± 2, and 31 ± 2%, and APD_90_ by 7 ± 1, 21 ± 2, and 29 ± 2%, respectively (Fig. 3, C and D).

**Figure 4:**
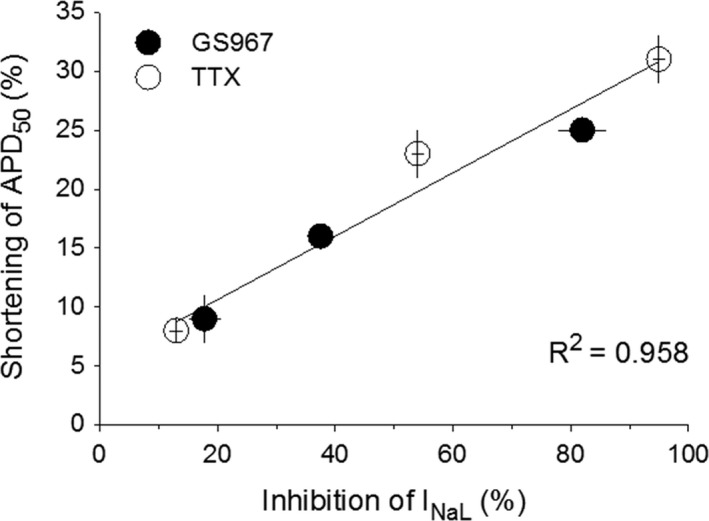
Correlation of APD shortening and *I*_N__aL_ inhibition in the presence of GS967 (●) and TTX (○). The percentage shortenings of APD are plotted against the percentage inhibition of *I*_N__aL_ caused by GS967 and TTX at the same concentrations. Coefficient of determination (*R* ^2^) is calculated using SigmaPlot linear regression curve analysis.

There was a close correlation (*R* ^2^ = 0.958) between the percentage inhibition of basal *I*_NaL_ and the percentage shortening of APD caused by either GS967 or TTX (Fig. 4), indicating that basal *I*_NaL_ significantly contributes to the APD.

### Inhibition of basal *I*_NaL_ by Na_V_1.5 channel blocker

**Figure 5:**
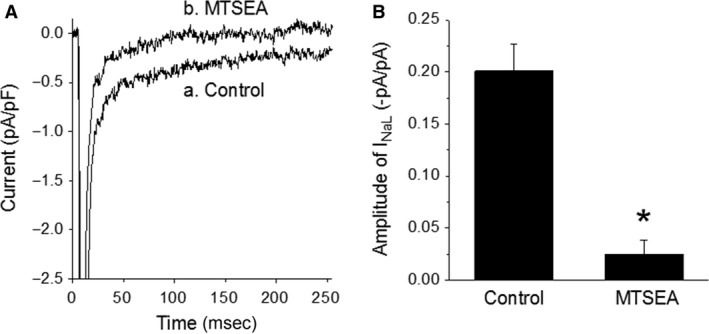
Inhibition of *I*_N__aL_ by the selective Na_V_1.5 channel blocker MTSEA (2 mmol/L). Panel A, currents recorded from a myocyte before (control) and after application of MTSEA. Panel B, summary of the results obtained from experiments shown in panel A. Bars represent the average amplitude of *I*_N__aL_ determined from 12 myocytes isolated from six hearts. *P < 0.001 versus control.

MTSEA is a selective blocker of Na_V_1.5 channels (Haufe et al. 2005; O'Reilly and Shockett 2012). In this study, MTSEA (2 mmol/L) was added to the bath solution to determine whether the basal *I*_NaL_ of myocytes was generated from the Na_V_1.5 channels. *I*_NaL_ was activated by depolarizing pulses from −90 to −30 mV. In this series of experiments, MTSEA decreased the amplitude of *I*_NaL_ by 90 ± 5%, from −0.20 ± 0.03 to 0.03 ± 0.01 pA/pF (*n* = 12/6, *P* < 0.001; Fig. 5). The result suggests that under the experimental conditions, the Na_V_1.5 channel is the major contributor to the *I*_NaL_ of guinea pig ventricular myocytes.

### Decrease in basal *I*_NaL_ by CaMKII inhibitors

Activation of CaMKII was reported to slow sodium channel inactivation. We used the CaMKII inhibitors KN‐93 and AIP, and an inactive analog of KN‐93 and KN‐92, as a negative control, to determine whether CaMKII plays a significant role in maintaining basal *I*_NaL_. The three drugs were applied through the recording pipette solution to three separate groups of myocytes, respectively.

**Figure 6:**
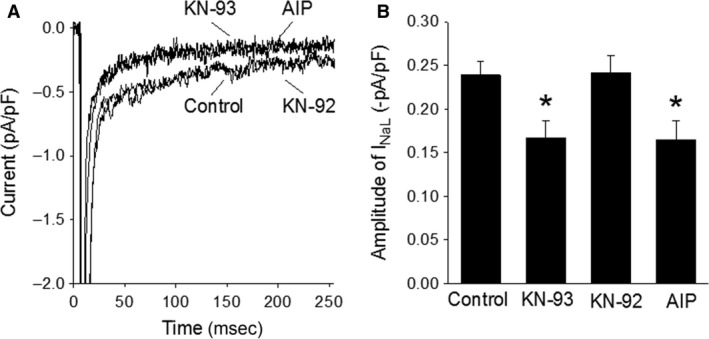
Decrease in *I*_N__aL_ in the presence of CaMKII inhibitors. Panel A, representative current traces recorded from four myocytes treated with no drug (control), KN‐93 (10 *μ*mol/L), KN‐92 (10 *μ*mol/L), and AIP (2 *μ*mol/L), respectively. Panel B, summary of the results obtained from experiments shown in panel A. Bars represent the average amplitude of *I*_N__aL_ in control (*n* = 40/17) and in the presence of KN‐93 (*n* = 11/3), KN‐92 (*n* = 12/4), and AIP (*n* = 11/5). *P < 0.05 versus control.

*I*_NaL_ was activated by voltage‐clamp pulses from −90 to −30 mV. The amplitude of *I*_NaL_ measured in the absence of drugs was −0.24 ± 0.02 pA/pF. KN‐93 (10 *μ*mol/L) and AIP (2 *μ*mol/L) reduced the *I*_NaL_ to −0.17 ± 0.02 pA/pF (*n* = 11/3, *P* < 0.05) and −0.17 ± 0.02 pA/pF (*n* = 11/5, *P* < 0.05), respectively (Fig. 6), whereas KN‐92 (10 *μ*mol/L) had no effect on *I*_NaL_ (−0.24 ± 0.02 pA/pF, *n* = 12/4; Fig. 6).

## Discussion

This study revealed that the basal *I*_NaL_ is of sufficient magnitude to affect the duration of the action potential of ventricular myocytes isolated from healthy guinea pigs. In the presence of the *I*_NaL_ blocker GS967 or low concentrations of TTX, the reduction in *I*_NaL_ was closely correlated with the shortening of APD (Figs. 2, 3, 4). Furthermore, the study showed that the basal *I*_NaL_ of guinea pig ventricular myocytes was mainly generated from the Na_V_1.5 channels (Fig. 5) and was regulated by CaMKII (Fig. 6). Thus, the results of the present study suggest that the basal, CaMKII‐mediated Na_V_1.5 I_NaL_ is a significant and physiological contributor to the action potential duration of guinea pig ventricular myocytes.

The action potentials of cardiac ventricular myocytes are characterized by a prominent plateau phase (phase 2) (Draper and Weidmann 1951). Repolarization is delayed during the plateau phase, and thus the duration of a myocardial action potential is largely determined by the length of the plateau phase. The action potential plateau is caused by a balance between depolarizing currents, including the inward L‐type (*I*_CaL_) and T‐type (*I*_CaT_) Ca^2+^ current, Na^+^–Ca^2+^ exchange current (*I*_NCX_) and *I*_NaL_, and repolarizing currents, such as the outward delayed rectifier K^+^ current (*I*_K_) (Ten Eick et al. 1992). The amplitude of *I*_NaL_ under physiological conditions is relatively small. In this study, the average amplitude of *I*_NaL_ was −0.24 ± 0.02 pA/pF. However, because *I*_NaL_ remains activated throughout the plateau phase and the membrane resistance is known to be high at the plateau (Weidmann 1951), even a small inward current such as *I*_NaL_ could play a significant role in maintaining cardiac depolarization, and thereby the duration of action potential. Myocytes used in this study were isolated from the whole ventricles; therefore, the present results represented the average effect of basal *I*_NaL_ on the duration of ventricular action potentials. As it has been reported that the density of *I*_NaL_ is greater in the mid‐myocardium than that in the epi‐ and endomyocardium (Zygmunt et al. 2001), it will be interesting to know if there is a regional difference in the contribution of *I*_NaL_ to the APD among different parts of ventricular myocardium.

Assessment of the contribution of basal *I*_NaL_ to APD requires the use of an inhibitor that, at least at certain concentrations, selectively and concentration dependently reduces *I*_NaL_, and has no effect on other ion currents that can modulate the APD. We used the *I*_NaL_ inhibitor GS967 at concentrations and conditions in which its inhibition was selective for the *I*_NaL_ (Belardinelli et al. 2013). For comparison, the Na^+^ channel blocker TTX applied at low concentrations was used to confirm that the inward current recorded was a Na^+^‐channel current. The selectivity of GS967 to inhibit *I*_NaL_ has been studied using rabbit ventricular myocytes (Belardinelli et al. 2013). The results of that study showed that, at a holding potential of −120 mV and a stimulation frequency of 0.1–3 Hz, GS967 (0.1–5 *μ*mol/L) concentration dependently blocked ATX‐II‐stimulated *I*_NaL_ without reducing the peak *I*_Na_. In addition, GS967 (1–3 *μ*mol/L) had no significant effect on *I*_CaL_, *I*_CaT_, and ATP‐sensitive K^+^ current, although GS967 at a high concentration of 10 *μ*mol/L caused a small (17%) inhibition of the rapid component of *I*_K_. In this study of guinea pig ventricular myocytes, GS967 concentration dependently inhibited ATX‐II‐induced *I*_NaL_ (Fig. 1), further confirming that this compound is a suitable pharmacological tool to investigate the role of *I*_NaL_ in cardiac repolarization. GS967 at 1 *μ*mol/L blocked the ATX‐II stimulated and the basal *I*_NaL_ by 100% and 82 ± 4%, respectively. Thus, it appears that the potency of GS967 to inhibit *I*_NaL_ is greater in the presence, than in the absence, of ATX‐II. This could be due to a sensitization by ATX‐II of sodium channels to the inhibitory action of GS967, as it has been found that sodium channel site‐3 toxins (such as ATX‐II) can enhance the binding and action of site‐1 toxin (such as TTX) and local anesthetics on this channel (Nishio et al. 1991).

A contribution of basal *I*_NaL_ to the APD was suggested by a previous study (Kiyosue and Arita 1989). In that study, TTX at a concentration of 60 *μ*mol/L caused a decrease in APD of ventricular myocytes isolated from healthy guinea pigs. However, TTX at such a high concentration could block not only the peak *I*_Na_, but also the L‐ and T‐type Ca^2+^ channels (Sun et al. 2008; Hegyi et al. 2012), which would also lead to a shortening of the APD. To verify the role of *I*_NaL_ in modulation of APD, we used the selective *I*_NaL_ blocker GS967 and low concentrations of TTX to determine the effect of an inhibition of basal *I*_NaL_ on the APD. Our results showed that GS967 and TTX at a concentration as low as 0.01 *μ*mol/L and 0.1 *μ*mol/L, respectively, could cause a significant shortening of the APD (Fig. 3). Furthermore, a quantitative analysis indicated that the inhibition of basal *I*_NaL_ and the shortening of APD caused by GS967 and TTX were closely correlated (Fig. 4).

Na_V_1.5 channel has been recognized as the dominant sodium channel of ventricular myocytes (Gellens et al. 1992; Maltsev et al. 2008; Veerman et al. 2015). In addition to the Na_V_1.5 channel, other sodium channel isoforms may also contribute to the cardiac sodium current. One study reported that A‐803467, a Na_V_1.8 channel blocker, blocked *I*_NaL_ of mouse and rabbit ventricular myocytes, suggesting that Na_V_1.8 channel contributes to cardiac *I*_NaL_ ((Yang et al. 2012). In contrast, another study found that A‐803467 had no effect on sodium current of mouse ventricular myocytes (Verkerk et al. 2012). In this study, the selective Na_V_1.5 channel blocker MTSEA (Haufe et al. 2005; O'Reilly and Shockett 2012) decreased the amplitude of basal *I*_NaL_ by 90 ± 5%, indicating that under the conditions of our experiments, the Na_V_1.5 channel isoform is a major contributor to basal *I*_NaL_ of guinea pig ventricular myocytes.

Cardiac myocytes overexpressing CaMKII showed an enhanced *I*_NaL_ (Wagner et al. 2006). In this study, we investigated the role of CaMKII in regulating basal *I*_NaL_ by comparing the amplitude of *I*_NaL_ in the absence and presence of the CaMKII inhibitor KN‐93 or AIP. Because KN‐93 and its inactive analog KN‐92 may have CaMKII‐independent effects on ion channels if applied extracellularly (Rezazadeh et al. 2006), these drugs and AIP were applied intracellularly through the pipette solution. Our results showed that the amplitude of basal *I*_NaL_ was decreased by either KN‐93 or AIP, but not by KN‐92, suggesting a significant role of CaMKII in regulating cardiac *I*_NaL_ under basal conditions (Fig. 6). However, CaMKII phosphorylation may not be the only mechanism to maintain basal *I*_NaL_. Other mechanisms, such as protein kinase C (Ma et al. 2012), may also be involved in the regulation of basal *I*_NaL_.

In summary, in this study we investigated the role of basal *I*_NaL_ in modulating the cardiac APD, by quantitatively determining the relationship between the amplitude of *I*_NaL_ and the duration of action potential. The results showed a close correlation between a decrease in *I*_NaL_ and a shortening of the APD, and thus provide strong evidence that basal endogenous *I*_NaL_ is a significant contributor to the APD of cardiac myocytes. The present results also demonstrated that the basal *I*_NaL_ of guinea pig ventricular myocytes is mainly generated from Na_V_1.5 channel isoform and is regulated by CaMKII.

## Conflict of Interest

Y. Song received a research grant from Gilead Sciences; L. Belardinelli was an employee of Gilead Sciences at the time of this study.
